# Advanced and Innovative Nano-Systems for Anticancer Targeted Drug Delivery

**DOI:** 10.3390/pharmaceutics13081151

**Published:** 2021-07-27

**Authors:** Lu Tang, Jing Li, Qingqing Zhao, Ting Pan, Hui Zhong, Wei Wang

**Affiliations:** 1 State Key Laboratory of Natural Medicines, Department of Pharmaceutics, School of Pharmacy, China Pharmaceutical University, Nanjing 210009, China; lutang@stu.cpu.edu.cn (L.T.); Linng@stu.cpu.edu.cn (J.L.); zhaoquincy@stu.cpu.edu.cn (Q.Z.); panting@stu.cpu.edu.cn (T.P.); 2 NMPA Key Laboratory for Research and Evaluation of Pharmaceutical Preparations and Excipients, China Pharmaceutical University, Nanjing 210009, China; 3 State Key Laboratory of Natural Medicines, School of Traditional Chinese Pharmacy, China Pharmaceutical University, Nanjing 211198, China

**Keywords:** nanotechnology, encapsulation strategy, targeted drug delivery, cancer therapy, chemotherapeutics, biopharmaceutics

## Abstract

The encapsulation of therapeutic agents into nano-based drug delivery system for cancer treatment has received considerable attention in recent years. Advancements in nanotechnology provide an opportunity for efficient delivery of anticancer drugs. The unique properties of nanoparticles not only allow cancer-specific drug delivery by inherent passive targeting phenomena and adopting active targeting strategies, but also improve the pharmacokinetics and bioavailability of the loaded drugs, leading to enhanced therapeutic efficacy and safety compared to conventional treatment modalities. Small molecule drugs are the most widely used anticancer agents at present, while biological macromolecules, such as therapeutic antibodies, peptides and genes, have gained increasing attention. Therefore, this review focuses on the recent achievements of novel nano-encapsulation in targeted drug delivery. A comprehensive introduction of intelligent delivery strategies based on various nanocarriers to encapsulate small molecule chemotherapeutic drugs and biological macromolecule drugs in cancer treatment will also be highlighted.

## 1. Introduction

Despite the rapid development of diagnostic and treatment strategies, cancer remains a leading cause of death worldwide and threatens the public health severely [1]. Cancer is a complicated disease condition that can spread to many parts of the body in an uncontrolled stage after onset [2]. The complexity of the carcinogenesis process limits the treatment regimens and requires a more rigorous and comprehensive therapeutic plan. Even though various new treatment modalities, such as immunotherapy, phototherapy, gene therapy and hormone therapy, are emerging; however, surgical intervention, radiation and, in particular, chemotherapy, continue to be the first line treatment option for most cancer patients [3]. Conventional chemotherapy is highly nonspecific in targeting the drugs to the cancer cells and can simultaneously kill healthy cells and cause systemic toxicity to the patients [4]. Moreover, several frequently encountered challenges of chemotherapeutics, including poor aqueous solubility, inadequate drug concentration at the lesion site, nonspecific biodistribution, intolerable cytotoxicity and the development of multiple drug resistance, severely limit the therapeutic efficacy and cause undesirable side effects [5]. Thus, the quest for innovative technologies remains an urgent necessity.

The application of nanotechnology to deliver anticancer agents has attracted growing interest for cancer treatment. The construction of nanosized drug delivery systems possesses tremendous potential due to their ability to improve the solubility of poorly soluble drugs and to reduce metabolism by dissolving them in their hydrophobic or hydrophilic compartment [6]. In addition, nanomedicine holds the advantages of passive targeting ability due to an enhanced permeability and retention (EPR) effect, a large surface-to-volume ratio for drug loading, a tunable size for modification, a prolonged plasma half-life and a different biodistribution profile compared to conventional chemotherapy [7]. Encouragingly, several chemotherapeutics-related nano-based formulations have been approved by the FDA for clinical applications, indicating the promising future of nanomedicine [8].

**Figure 1:**
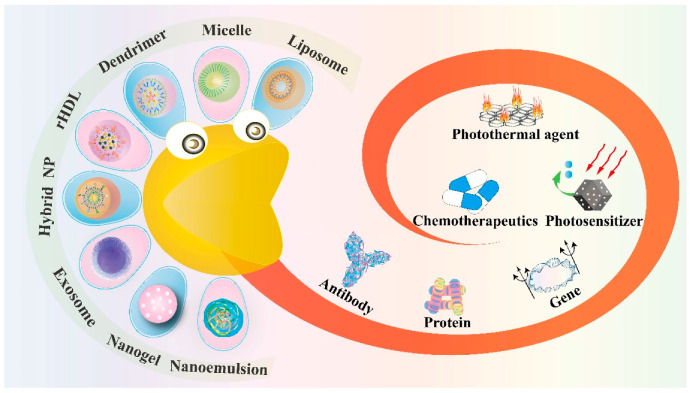
Schematic illustration of novel nano-encapsulation based on various drug delivery vehicles.

Typical nano-based delivery vehicles include liposome, micelle, dendrimer, inorganic vector, nanogel and nanoemulsion, while novel nanocarriers also contain biomimetic reconstituted high-density lipoprotein (rHDL), exosome and the hybrid nanoparticle, which come from the mixture of nanomaterials [6]. Each of these nanotools displays its unique physiochemical properties and possesses the ability for further modification of active targeting ligands. Therefore, this review focuses on the application of nanotechnology in cancer therapy and discusses how these nanoparticles (NPs) encapsulate the therapeutic agents in targeted drug delivery (Figure 1). Table 1 is a summary of all these nanostructures discussed below and their corresponding encapsulation of anticancer agents.

## 2. Liposome

**Figure 2:**
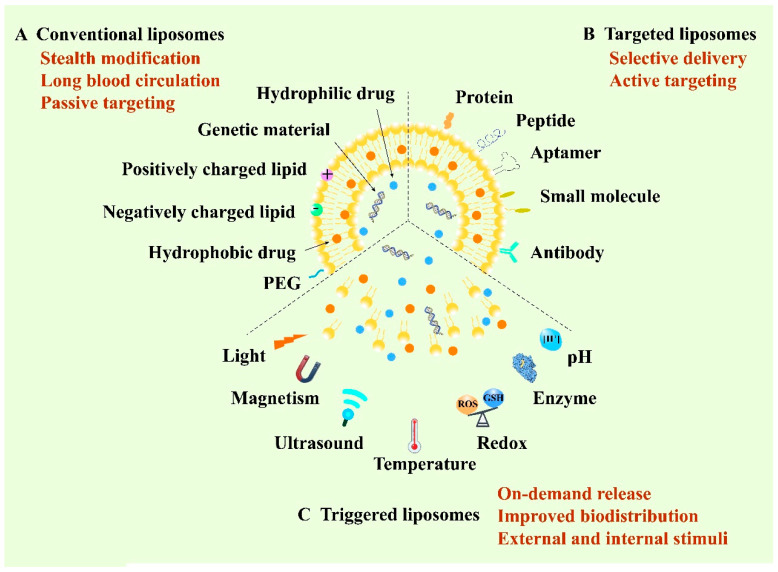
Schematic illustration of various liposome-based delivery systems and their representative properties and typical functions.

Liposome, structured by natural non-toxic phospholipids and cholesterol, is a spherical vesicle containing an aqueous inner compartment and a phospholipid bilayer, which is able to load both hydrophilic and hydrophobic cargos [43,44]. Structurally, the similar composition with the biological membrane allows cells to take up liposomes more easily and cause lower toxicity [45]. Liposomes represent one of the most promising delivery vehicles for anticancer therapy owing to their biocompatibility, high loading capacity and modifiable sites [46]. So far, they are the most widely developed nanomedicines and are also the most likely to be put onto the market [47]. According to the development of liposomes, they can be classified into conventional liposomes, targeted liposomes and triggered liposomes. The first conventional liposomes mainly adopt the stealth strategy to escape capture by the reticuloendothelial system (RES) and to prolong blood circulation [48]. Polyethylene glycol (PEG) is the most widely used material for the stealth modification of the liposomes, thus the PEGylated liposomes are also called stealth liposomes [49]. Furthermore, stealth liposomes can achieve passive targeting by virtue of the EPR effect [50]. In 1995, Doxil^®^, as the first stealth liposome encapsulating doxorubicin (DOX), was approved by the FDA for the clinical application of cancer treatment [51]. However, recent research has reported that PEGylated liposomes can elicit unexpected immune responses, such as the accelerated blood clearance (ABC) phenomenon [52]. In this case, the long-circulating properties of PEGylated liposomes will decrease when administered repeatedly at certain intervals to the same animal [53]. To achieve selective delivery, targeted liposomes were developed by modifying various active targeting ligands including antibodies, aptamers, small molecules and peptides to the surface of the membrane of liposomes to target the specific receptors of tumor cells [54,55]. Recently, stimuli-responsive liposomal systems have emerged as an attractive approach for the on-demand release of encapsulated drugs. This kind of liposome can respond to specific stimuli, either external stimuli such as light, temperature, ultrasound and magnetism, or internal stimuli such as pH, enzyme and redox [56,57]. Additionally, liposomes can co-load multiple drugs with different structures, ranging from small molecules to macromolecules such as proteins and genes, for combination administration to improve the therapeutic efficacy [58]. Therefore, liposomes play a vital role in anticancer targeted drug delivery and are of great research and clinical interest (Figure 2).

### 2.1. Encapsulation of Small Molecule Drugs with Liposome

IOX1 (5-carboxy-8-hydroxyquinoline), a kind of histone demethylase inhibitor, is used as an antibody-free small molecular drug for immunotherapy. Liu et al. fabricated IOXL by loading IOX1 into liposome with a similar formula of commercially available PEGylated liposomal DOX (PLD, LIBOD^®^) [9]. Subsequently, the mixed liposome IPLD was obtained by mixing IOXL with commercial PLD at an optimized molar ratio. DOX can induce immunogenic the cell death (ICD) of cancer cells and promote the transfer of tumor-associated antigens to dendritic cells (DCs), and thus activate DC maturation and the infiltration of T cells and memory T cells to the tumors. Synergistically, IOX1 could inhibit cancer cells’ P-glycoproteins (P-gp) through the JMJD1A/β-catenin/P-gp pathway and greatly enhance DOX-induced immune-stimulatory ICD. As a result, the IOX1 and DOX combination greatly promoted T cell infiltration and activity and significantly reduced tumor immunosuppressive factors. Therefore, long-term antitumor immunities were observed after the treatment of IPLD in murine colon cancer CT26 cells. In addition, the antitumor performance of IPLD was better than that of the combination of DOX with anti-PD-L1 antibody (αPD-L1) against subcutaneous (*s.c.*) CT26 tumors in BALB/c mice without apparent adverse effects.

Mitochondria, one of the vital intracellular organelles, play a crucial role in cellular metabolism and serve as key regulators of cell death [59]. Hence, mitochondrial targeting has been widely explored as a supplementary method to induce cancerous cells’ ablation [60]. Owing to the negative charge of the mitochondrial membrane, lipophilic triphenylphosphonium (TPP) cation is commonly modified to various nanocarriers for mitochondrial targeting [61]. Jiang et al. fabricated a novel mitochondrion-targeted liposome based on dendritic lipopeptide (DLP) modified with arginine residues, showing a 3.7-fold higher level of accumulation in the mitochondria of 4T1 cells than that of a TPP decorated nanoplatform [10]. The encapsulated photosensitizer indocyanine green (ICG) was also delivered into the mitochondria of the tumor cell, resulting in complete tumor eradication in mice bearing 4T1 mammary tumors after photo-irradiation.

To enhance the efficacy of oxygen-dependent PDT, platinum NPs (nano-Pt), acting as catalase (CAT)-like nanoenzymes, can generate oxygen through catalysis of elevated H_2_O_2_ in cancer cells [62,63]. An example was designed by Liu et al.; they adopted a reverse phase evaporation strategy to improve the aqueous drug loading capacity of nano-Pt in the liposome; then, the clinical hydrophobic photosensitizer verteporfin (VP) was loaded into the lipid bilayer to confer PDT activity. Finally, murine macrophage cell membranes were hybridized into the liposomal membrane to endow the delivery system with biomimetic and targeting features. The resulting liposomal system, termed nano-Pt/VP@MLipo, exhibited a long circulation time and inflammatory endothelium targeting ability [11]. After targeting to the tumor site, the self-supply of oxygen improved the VP-mediated PDT effect, which in turn triggered the release of nano-Pt via membrane permeabilization. Under light irradiation, nano-Pt/VP@MLipo showed remarkable tumor inhibition in 4T1 tumor-bearing BALB/c mice, which also inhibited the lung metastasis and extended the survival time without overt toxicity.

### 2.2. Encapsulation of Biological Macromolecules with Liposome

As an essential constituent of the electron transport chain in mitochondrion, the apoptotic protein cytochrome C (CytoC) can mediate the initiation of cell apoptosis after being transported to the cytoplasm of cancer cells [64,65]. Chen et al. designed a liposome-based nanoassembly (p53/C-rNC/L-FA) for intracellular site-specific delivery of an apoptotic protein CytoC and a plasmid DNA encoding tumor-suppressing p53 protein (p53 DNA) [12]. p53/C-rNC/L-FA consisted of an acid-activated fusogenic liposomal membrane shell modified with folic acid (L-FA) and a DNA/protein complex core assembled by the p53 DNA, protamine and CytoC-encapsulated redox-responsive nanocapsule (C-rNC). With the favorable tumor-targeting capacity of FA, p53/C-rNC/L-FA achieved a high level of accumulation in the tumor that overexpresses the folate receptor in vivo. Owing to the arginine-rich nucleus-targeted protamine, the p53 DNA could efficiently accumulate in the nucleus and produce the p53 protein for tumor suppression, which, in combination with the pro-apoptotic effect of CytoC, could augment anticancer efficacy. In vivo antitumor activity of p53/C-rNC/L-FA showed great potential for inducing tumor cell apoptosis and inhibiting tumor growth in the orthotopic MCF-7 tumor mice model.

To overcome the tumor hypoxia, exogenous H_2_O_2_ and its decomposing catalase (CAT) were separately loaded into the PEG modified stealthy liposomes by Song et al. [13]. In vivo results demonstrated that the combined treatment of CAT@liposome and H_2_O_2_@liposome could promote tumor oxygenation, which further reversed the polarization of immune-supportive M1-type tumor-associated macrophages (TAMs). Due to the relieved tumor hypoxia, the favorable antitumor immunities were formed, which remarkably enhanced the tumor suppression efficacy by radiotherapy (RT) to promote the infiltration of cytotoxic T lymphocytes (CTLs) and benefited the further application of the CTLA-4 checkpoint blockade to inhibit tumor growth. The striking radio-immunotherapy was achieved both in 4T1 tumor-bearing mice models and prostatic patient-derived xenograft (PDX) tumor models. Moreover, no additional toxic side effects were observed during the treatment.

## 3. Reconstituted High-Density Lipoprotein (rHDL)

High-density lipoprotein (HDL) is an important member of the lipoprotein family, which possesses multiple functions. HDL is a type of serum protein that is mainly composed of apolipoprotein A-I (apoA-I), phospholipids, free cholesterol, cholesteryl ester (CE), triglyceride (TG), and other components [66,67,68]. There are many distinctions among different types of HDL, which can be classified according to hydration density, particle size, charge and composition of apoA. In addition to apolipoprotein, there are other types of proteins in HDL, such as lecithin cholesterol acyltransferase (LCAT), cholesteryl ester transfer protein (CETP), apolipoprotein M (apoM) and paraoxonase (PON) [69,70]. Interestingly, many studies have shown that, with the increase of the proportion of proteins in HDL, the molecular weight of HDL will also increase. HDL is expected to become a promising delivery candidate for anticancer therapy owing to its good biocompatibility, fewer side effects, lower toxicity and the characteristics of reversing cholesterol transport, anti-oxidation and anti-inflammation [71]. However, the complex composition, the strict extraction process and the low yield greatly limit the application scope of HDL. Hence, to overcome the above tricky problems and retain the excellent properties of HDL, reconstituted high-density lipoprotein (rHDL) is manually synthesized using various common materials in vitro.

**Figure 3:**
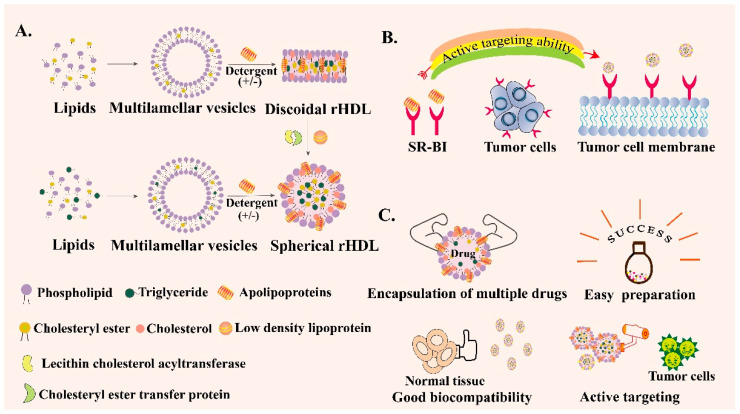
Schematic introduction of reconstituted high-density lipoprotein (rHDL). (**A**) Preparation process of spherical rHDL and discoid rHDL. (**B**) Active targeting ability of rHDL through scavenger receptor class B type I (SR-BI)-mediated specific targeting. (**C**) Various advantages of rHDL as a drug delivery vehicle.

rHDL can be synthesized and its properties are similar to HDL [72]. In terms of composition, they both contain apoA-I, which accounts for about 65% of HDL and 75% of rHDL. rHDL has two configurations, spherical rHDL (s-rHDL) and discoid rHDL (d-rHDL) [73]. Compared with HDL’s complex constituents, rHDL generally contains apoA-I, phospholipids, free cholesterol, CE, TG, or sometimes only includes the first three components (Figure 3A). In addition, the content of each component can be flexibly adjusted according to the experimental purpose. Scavenger receptor class B type I (SR-BI) is overexpressed in tumor cells and participates in the cholesterol metabolism of tumor cells [74,75]. ApoA-I can specifically target SR-BI and mediate the anti-inflammatory ability of macrophages by regulating the production of cytokines in macrophages [76]. Therefore, SR-BI can be used as a specific target for the treatment of common tumors such as breast cancer, ovarian cancer and prostate cancer [77,78] (Figure 3B). Hence, rHDL can be regarded as an outstanding encapsulated carrier to deliver various drugs with the following unique advantages (Figure 3C). First of all, rHDL-based NPs possess a small particle size and SR-BI targeting ability so that they can actively orientate to the tumor site. Secondly, they have good biocompatibility and low toxicity and side effects. In addition, no obvious drug leakage is identified in the rHDL encapsulated delivery system, which is suitable for enhancing the stability and solubility of some insoluble drugs, changing the pharmacokinetic parameters, and prolonging the circulation time [79]. Besides, rHDL itself can also exert a direct effect on the tumor cells; the main component apoA-I exhibits anti-neoplastic biological effects indirectly via alterations in the functions of macrophages and other immune cells in vivo [80]. Therefore, to overcome the physicochemical limitations of free drugs, including poor solubility and instability, decreasing immune clearance, off-target deposition and other disadvantages, exploring the bio-derived NPs like rHDL is necessary [81]. To summarize, rHDL is a new type of biomimetic nanocarrier that has been emerging in recent years. rHDL has many excellent characteristics such as the capability to overcome several biological barriers in cancer therapy, intrinsic targeting ability, endosomal escape ability, high biological safety and so on. However, the complex preparation process may restrict its wide clinical application and scale-up preparation. Therefore, more and more studies need to be carried out to simplify the preparation method of rHDL, making it easy to clinically translate.

### 3.1. Encapsulation of Small Molecule Drugs with rHDL

Cell-penetrating peptides (CPPs), cationic or amphipathic in nature, can facilitate efficient translocation across the cell membrane. However, the application of CPPs is badly restricted because of the extensive penetration in vivo and the poor selectivity and permeability before reaching target sites [82]. Gambogic acid (GA) can activate impaired apoptosis pathways in cancerous cells through a complex mechanism involving the participation of multi-molecular targets. In addition, GA can serve as a p53 inducer to stimulate cancer cell apoptosis [83]. Ding et al. designed nano-encapsulated NPs, which consisted of GA-loaded lipid NP (LNP/GA) as a lipid core, then they inserted apoA-I and a pH-responsive CPP (STRR6H4) onto the surface of the LNP to form GA-encapsulated and CPP-anchored rHDL (cp-rHDL/GA) [14]. In vitro and in vivo results based on the HepG2 xenograft mice model showed that the new design had an outstanding antitumor effect with remarkable cytotoxicity and apoptotic effect via triggering the p53 pathway, exhibiting an approximately 5-fold increase in IC_50_ compared to free GA, indicating the promising future of this biomimetic nanoplatform in drug encapsulation.

Valrubicin is a kind of chemotherapeutic drug that is mainly used for bladder cancer treatment [84]. Sabins et al. developed a novel targeting approach through the encapsulation of valrubicin into a superparamagnetic iron oxide nanoparticle (SPION) containing rHDL to form rHDL-SPION-valrubicin hybrid NPs by the self-assemble method [15]. The experimental results showed that the solubility and bioavailability of valrubicin were greatly enhanced after encapsulation into rHDL NPs. The cytotoxicity test toward PC-3 cells demonstrated that rHDL-SPION-valrubicin NPs were up to 4.6 and 31 times more effective at the respective valrubicin concentrations of 42.4 µg/mL and 85 µg/mL than valrubicin alone, implying that rHDL-based targeted drug delivery through magnetic navigation could effectively enhance the therapeutic efficacy of prostate cancer treatment.

### 3.2. Encapsulation of Biological Macromolecules with rHDL

Small interfering RNA (siRNA) has shown enormous potential in cancer treatment because it can silence specific gene expressions, such as vascular endothelial growth factor (VEGF), of which expression can be up-regulated in response to hypoxia in ischemic tumor tissues [85]. With this aim in mind, Ding et al. developed nanocomplexes consisting of fluorescent-tagged apoA-I and cholesterol-conjugated siRNA (Chol-siRNA) to form Chol-siRNA-loaded rHDL NPs (rHDL/Chol-siRNA), providing an effective approach to transfer Chol-siRNA across the membrane directly into the cytoplasm via the SR-BI-mediated non-endocytotic mechanism, which bypassed endo-lysosomal trapping [16]. In addition, the new nanocomplexes based on rHDL significantly enhanced antitumor efficacy against MCF-7 human breast cancer in vivo, efficiently decreased VEGF expression level and inhibited the formation of intratumoral microvessels in the tumor tissue.

Except for siRNA, the deregulation of microRNAs (miRNAs) also involves in tumor angiogenesis and thus offers an opportunity for exploring new therapeutic approaches to modulate angiogenesis [86]. However, the lack of effective miRNA delivery strategies limits its practical application. To overcome this obstacle, Chen et al. designed miR-204-5p inhibitor (miR204-5p-inh) encapsulated rHDL NPs to suppress tumor growth in the HeyA8-MDR ovarian tumor-bearing mice model [17]. This novel NP could arrive at the tumor site by binding with SR-BI. The in vivo and in vitro results revealed that miR inhibitors could be delivered by rHDL NPs to silence the expression of the oncogene miR-204-5p at tumor sites, opening rational avenues for solving issues in tumor anti-angiogenic therapy.

## 4. Micelle

Micelles refer to a large number of molecular-ordered aggregates that begin to form when the concentration of surfactant reaches a concentration above the critical micelle concentration (CMC) in an aqueous solution [87]. In micelles, the hydrophobic group of the surfactant molecule aggregates to form the core, and the hydrophilic polar group forms the outer layer of the micelles [88]. The compounds that form micelles are generally amphiphilic molecules, so the micelles are not only soluble in polar solvents, such as water, but also non-polar solvents in the form of reverse micelles [89]. Because of the above characteristics, micelles can be used as a drug delivery carrier. Plenty of antitumor drugs have poor water solubility and bad stability, which limit their broad application in cancer therapy. However, the hydrophobic blocks that constitute the core compartment of polymeric micelles can load those hydrophobic drugs effectively. Generally, there are two methods to encapsulate drugs in polymer micelles; the first is to encapsulate the hydrophobic drug in the hydrophobic region of the micelle through physical action, and the other is to connect the drug to the hydrophobic core through chemical bonds, different encapsulation methods can be designed according to the properties and experimental purpose of certain drugs [90,91].

Micelles have various shapes including spherical, layered, rod-shaped, worm-like, and hexagonal shapes [92]. According to the length of the hydrophobic block, micelles can be divided into two types. If the length of the hydrophilic end is longer than the specific hydrophobic end, a star micelle will be formed, otherwise, a crew-cut micelle will be formed [93]. Most of the copolymer micellar hydrophilic blocks are biocompatible copolymers such as PEG, polyethylene oxide (PEO), and polyvidone (PVP). Hydrophobic blocks are biodegradable copolymers such as polylactic acid (PLA), poly (lactic-co-glycolic acid) (PLGA) and polycaprolactone (PCL). There are also non-degradable copolymers, such as polystyrene (PS), and triblock hydrophilic–hydrophobic copolymers, such as micelle materials like poloxamer (PEO-PPO-PEO) and PEG-PLGA-PEG [94]. Currently, different kinds of micelles are designed for intelligent tumor-targeted drug delivery according to the properties of the tumor microenvironment such as low pH, hypoxia, high tissue osmotic pressure and the overexpression of matrix metalloproteinases. Therefore, the micelle-based nano drug delivery system is also a promising tool in anticancer targeted therapy.

In summary, micelles are easy to prepare and can encapsulate multiple drugs while protecting them against degradation and destruction. They are characterized by high stability, multiple available routes of administrations and the possibility of adjusting the controlled drug release with environment correspondence. Furthermore, compared to other nanomaterials, micelles have the feature of biodegradability, low immunogenicity and non-toxicity, which can be used as a safe carrier in drug delivery. Genexol-PM is a polymeric micellar formulation of paclitaxel without cremophor EL. Keam et al. conducted a Phase II Study that investigated the efficacy and safety of Genexol-PM plus cisplatin as induction chemotherapy (IC) in patients with locally advanced head and neck squamous cell carcinoma (LA-HNSCC) [95]. The results showed that IC with Genexol-PM and cisplatin exhibited the modest tumor response with well-tolerated toxicity profiles for patients with LA-HNSCC. Considering the high clinical transformation potential of micelles, it is very necessary to speed up the research on micelles.

### 4.1. Encapsulation of Small Molecule Drugs with Micelle

Artesunate (ART), a semi-synthetic derivative of artemisinin, has been reported by many studies to have antitumor effects on various cancer cell lines [96,97]. However, the applications of ART are limited due to its poor water solubility and stability, low bioavailability and short half-life in blood circulation. Therefore, it is desirable to design an ART depot with sustained release and targeted delivery properties. Hao et al. prepared a micelle composed of pH-sensitive PEOz as hydrophilic corona and polylactide/PBAE conjugation with ART as the hydrophobic core [18]. The outside copolymer exhibited proton sponge properties at a pH below its pKb (~6.5) owing to the presence of amine groups, which resulted in its pH sensitivity [98]. The micelles showed enhanced cellular internalization and a stronger inhibitory effect on CT-26 cells in vitro. In addition, the antitumor effect based on the colon carcinoma mice model also showed that the ART-based micelle group could decrease the most tumor volumes compared to the other groups.

S-nitrosoglutathione (GSNO) is a prodrug that can generate nitric oxide (NO) under the presence of glutathione (GSH) [99]. Many studies have revealed that NO has the potential to overcome multidrug resistance (MDR) with low side effects [100]. However, GSNO is unstable and its stability can be easily affected by ambient temperature, pH and other factors. Besides, NO is released rapidly in water, resulting in the limited application range of GSNO. The poly(propylene sulfide) (PPS) block is able to converse from hydrophobic to hydrophilic under the condition of excess ROS in the tumor sites, which enables the on-demand delivery of therapeutic agents [101]. Therefore, in order to retain the advantages of GSNO, while minimizing its disadvantages, Wu et al. chemically conjugated GSNO to an amphiphilic block copolymer PPS-PEG (GSNO-PPS-PEG), and then encapsulated the anticancer drug DOX in the region of PPS to form a spherical micelle [19]. An in vitro release study showed that the micelle was ROS- and GSH-responsive. To investigate the potential of PEG-PPS-GSNO in overcoming the MDR of cancer, the toxicity of free DOX and PEG-PPS-GSNO@DOX toward HepG2/ADR cells were tested and compared in vitro. As the experiment’s results showed, the micelle group exhibited the strongest cytotoxicity compared to the other groups, indicating that this nano drug delivery system (DDS) could serve as an effective co-delivery platform of NO and DOX to selectively kill chemo-resistant cancer cells.

Retinoblastoma (Rb) is a malignant tumor of the retina in infants and is also the most common childhood malignancy. Melphalan (MEL) can be regarded as an effective chemotherapeutic agent for the treatment of Rb. Li et al. adopted micelles as an ideal drug delivery vehicle to solve the MEL inherent cardiopulmonary toxicity and realize precision drug delivery [20]. N-acetylheparosan (AH) is a natural heparin-like polysaccharide in mammals with a long circulation effect and good biocompatibility which was linked by d-α-tocopherol acid succinate (VES) via cystamine (CYS) to synthesize reduction-responsive N-acetylheparosan-CYS-Vitamin E succinate (AHV) copolymers. Furthermore, MEL-loaded AHV (MEL/AHV) micelles were prepared with the properties of small particle size, high drug loading content, and obvious reduction-triggered release behavior. In vitro antitumor effects based on WERI-Rb-1 Rb cells showed that the MEL/AHV micelles could significantly enhance the cytotoxicity against Rb tumor cells, suggesting that MEL/AHV micelles might be a potential delivery system for the enhanced delivery of melphalan to Rb cells.

### 4.2. Encapsulation of Biological Macromolecules with Micelle

The desmoplastic and hypoxic microenvironment of pancreatic cancer can induce the aberrant expression of miRNAs and hypoxia-inducible factor-1 (HIF-1), which are responsible for gemcitabine (GEM) resistance [102]. MiR-519c could bind to HIF-1 mRNA and then inhibit HIF-1 expression, slow down the tumor metabolism and decrease profibrotic and angiogenic responses. Xin et al. used 2′-O-methyl phosphorothioate (2′-OMe-PS) to modify miRNA and formed OMe-PS-miR-519c, then they conjugated the complex to a kind of redox-sensitive polymeric micelle via stable disulfide bonds [21]. The effect of the micelle on the MIA PaCa-2R cells was determined in vitro, suggesting that miR-519c inhibited the tumor cells’ proliferation by inducing G2 phase cell cycle arrest and sensitizing GEM to kill hypoxic GEM-resistant tumor. Moreover, in vivo and in vitro results based on the desmoplastic pancreatic cancer mice model showed that the multifunctional nano-micelle could effectively deliver GEM and miR-519c to the tumor site and reverse the GEM resistance remarkably, which resulted in synergistic inhibition of pancreatic cancer.

Many studies have shown that the epigenetic regulation drug histone deacetylase inhibitor (HDACi) was able to reverse exhausted T cells by changing the epigenetic transcription program, which can solve the challenge of immune escape and be beneficial to PD-1/PD-L1 blockade therapy. Lu et al. developed a kind of micelle called PDDS to encapsulate the siRNA of PD-L1 to form pH-sensitive siRNA-PD-L1-loaded micelles (siRNA@PPDS) [22]. In vitro and in vivo results revealed that the complexes could not only be spontaneously released into the cytoplasm under the acidic and lipase conditions in the lysosome, thereby inducing B16-F10 melanoma tumor cells’ apoptosis, but also silence the expression of PD-L1 protein in a dose-dependent manner. Furthermore, the micelles also showed outstanding inhibition ability of pulmonary metastasis.

Encouragingly, in addition to the micelle-related pre-clinical studies mentioned above, many polymeric micelles have been developed as antineoplastic drugs, such as Genexol-PM^®^ and Paclical^®^. Besides, many other polymer micelle-based nanomedicines, including NK012, NK105, SP1049C and NC-4016, are under clinical evaluation for treating several types of tumors, indicating their bright future in clinical translation [103].

## 5. Dendrimer

**Figure 4:**
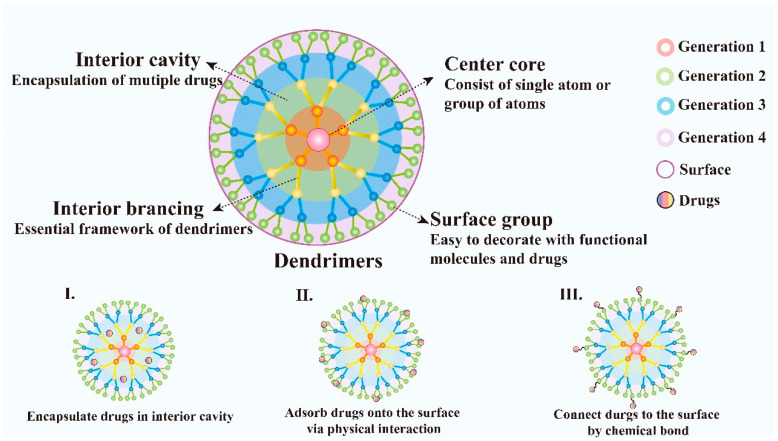
Schematic diagram of dendrimer architecture. Distinct functions of each dendrimer component and various ways of dendrimers to deliver drugs are also illustrated.

Dendrimers are novel polymeric materials with a 3D structure that has extensively branched. Dendrimers have multiple functional groups on the surface which makes them easy to be decorated and provides a new train of thought to its application. The dendrimers generally consist of a central core that includes a single atom or group of atoms, building blocks containing many layers of repeating units known as generations, and numerous functional groups on the surface, which play a key role in their properties [104] (Figure 4). Compared with traditional linear polymers, dendrimers have a variety of new physical properties because of their unique structure with numbers of chains whose ends are combined with a high degree of branching, these characteristics make them attractive for biological applications and drug delivery. Dendrimers possess many charming properties such as good water-solubility, nanoscale uniform size, symmetrical shapes, high densities of peripheral functionalities, available internal cavities, good biocompatibility, biosafety, stability and large drug loading capacity [105]. In addition, the structure of dendrimers is convenient to synthesize and the products with fixed molecular weight due to their step-by-step controlled synthesis are easy to obtain. They can be synthesized using divergent methods or convergent methods [106]. Dendrimers have been demonstrated as an ideal carrier for the targeted delivery of therapeutic and diagnostic agents, such as the delivery of small molecular chemotherapeutic drugs, macromolecular biological drugs, genes, proteins and enzymes [107]. In addition, dendrimers can be designed to realize controlled release by electrostatic interactions, conjugation of the drug to dendrimers, encapsulation of drugs within the dendritic architecture [108]. Moreover, the nanostructure of dendrimers facilitates the passive targeting of drugs to tumor tissues via the EPR effect. Besides, because of their immunogenicity, dendrimers can also be used as a carrier for gene delivery.

To conclude, dendrimers are different from linear polymers by architecture with tailor-made surface groups. Their properties are mainly determined by the functional groups on their surface with many modified methods. They have the advantages of biocompatibility, easy elimination from the body and appropriate particle size to express the EPR effect. The limitation of dendrimers is also the complicated synthesis process, which requires many chemical reactions. Besides, the cytotoxicity to normal cells resulting from the physiological stability of cationic groups of dendrimers may exert certain security threats. Fortunately, dendrimers have been applied in many fields since they came out, VivaGel^®^ is the first dendrimer-based commercial medical product and many others are now in clinical trials, indicating its promising future in anticancer therapy [109].

### 5.1. Encapsulation of Small Molecule Drugs with Dendrimer

Targeted delivery of small molecular chemotherapeutics to the tumor site is an effective means to achieve antitumor purposes. For example, DOXIL^®^ can effectively accumulate in the tumor tissues but fail to penetrate through the tumor, thus designing a DDS is essential to achieving the predicted goal [110]. Sun et al. made a cluster-bomb-like nanocarrier that synergized based on dendrimers. A sixth-generation nontoxic degradable polyaminoester dendrimer with a diameter of 5 nm was synthesized with pH-dependent 2-(*N*,*N*-diethylamino)ethyl termini. A fusogenic phospholipid DOPE (1,2-dioleoyl-*sn*-glycerol-3-phosphoethanol-amine) was chosen to coat the dendrimers, but this DOPE was expected to peel off by fusion once inside the tumor tissue. A PEGylated lipid DSPE-PEG and cholesterol were added to make the nano-assembly stealthy and stable in the blood [23]. The dendrimers’ core contained many tertiary amines and they were hydrophobic at a neutral pH but could become water-soluble at a lower pH condition. Hence, hydrophobic DOX could be encapsulated inside the dendrimers at a neutral pH and released once at a lower pH. The antitumor efficacy was tested based on human ovarian cancer cells SKOV-3 in vitro and the dendrimers were found to ship the drugs into the cytosol and circumvent the multidrug resistance. The in vivo therapeutic efficacy of this DDS was compared with the micellar PCL-PEG/DOX using subcutaneous drug-sensitive/resistant MCF-7 and MCF-7/ADR breast tumor-bearing mice, which showed that the DDS based on dendrimers had a superior antitumor effect.

Pyropheophorbide-a (Ppa) is a kind of photosensitizer that can produce ROS under laser irradiation, resulting in a high level of apoptosis of tumor cells. Therefore, Ppa could be used in PDT according to its unique optical property. Polyglutamic acid (PGA) is a type of natural anion polymer that is biodegradable and safe. Wang et al. synthesized a PGA-based glycosylated dendritic agent (PG-L8G-Ppa-Dendron, PGPD) to load Ppa to treat triple negative breast cancer (TNBC). The PGPD could self-assemble into spherical NPs and enable the successful encapsulation of Ppa [24]. In vitro and in vivo results based on the TNBC mice model showed that treatment with these nanocomplexes under laser irradiation produced a stronger antitumor effect than that with free Ppa. In addition, the dendrimers had a great tumor enrichment effect, leading to the efficient induction of ROS production and a significant inhibition of tumor growth.

The dense fibrotic stroma in pancreatic ductal adenocarcinoma (PDA) resists drug diffusion into the tumor and leads to an unsatisfactory prognosis [111]. To solve this thorny problem, Wang et al. designed a new polymer dendrimer based on the PAMAM dendrimer. They covalently conjugated camptothecin (CPT) to PAMAM dendrimers through an ROS-sensitive linker followed by surface modification with GSH [25]. The novel DDS could actively penetrate deep into PDA tumors through γ-glutamyl transpeptidase (GGT)-triggered cell endocytosis and transcytosis. BxPC-3 cells were used to test the tumor penetration ability of this DDS in vitro, and the results revealed that the DDS had ideal tumor penetration ability, which occurred via the GGT-activated cationization and was dependent on the caveolae-associated internalization and efflux process. In vivo antitumor activity based on BxPC-3 tumor-bearing nude mice demonstrated that this dendrimer-drug conjugate possessed a high efficiency of active tumor penetrating capability via transcytotic transport with ROS-responsive drug release for PDA therapy.

### 5.2. Encapsulation of Biological Macromolecules with Dendrimer

It is known that normal epithelial cells and epithelial tumor cells are different in the glycoprotein pattern of their outer cell membrane and there are some differences in the structure of the mucin between normal cells and tumor cells; therefore, transmembrane glycoprotein Mucin 1 (MUC1) is considered a potential target for cancer therapy [112]. Gaidzik et al. combined MUC1 glycopeptides with an immunostimulant Toll-like receptor 2 lipopeptide and T-cell peptide epitopes to override the natural tolerance and induce a sufficiently strong immune response [26]. The two types of immunostimulants were encapsulated into the cavity of the polymer dendrimers. The mice were immunized and found to produce corresponding antibodies in vivo. In addition, more than 95% of the tumor cells were recognized by the antibodies induced through the dendrimers and the breast tumor volumes were smaller compared to other therapy groups, indicating the effective vaccine delivery ability of dendrimers to tumor sites.

Lv et al. designed new nanocomplexes based on a bioreducible and amphiphilic dendrimer bearing a fluoroalkyl tail. The fluorolipid was conjugated to the focal point of a cysteamine-cored polyamidoamine dendrimer via a disulfide bond, while phenylboronic acid moieties were decorated on the dendrimer surface for efficient protein binding [27]. The novel nanocomplexes showed high protein binding capability and good stability and could efficiently release the cargo of a membrane-impermeable toxin protein saporin in a GSH-responsive manner. In addition, in vitro experiments showed that the polymer could efficiently deliver the protein into 4T1 breast cancer cells and caused a tumor killing effect. Besides, in vivo results demonstrated that the polymer could also inhibit tumor growth in the 4T1 tumor-bearing mice model.

## 6. Nanogel

Nanogel (NG), a type of systemic drug delivery carrier, is simply defined as particles of gel with 3D hydrophilic polymeric network structures similar to the internal structure of hydrogels [113]. NG can be fabricated through chemical and physical crosslinking or self-assembly with the interactions of the hydrophobic, electrostatic bonding between the polymers in heterogeneous colloidal environments [114]. Common materials to constitute NG include polyacrylic acid (PAA), polyacrylamides (PAM), pluronic, multi-carbohydrates, and other high molecular polymers containing hydrophilic functional groups, such as protein-polysaccharides, polyvinyl alcohol (PVA) and polyaminoacid [115]. Additionally, NG can be prepared through micro-molding and photolithographic methods, modification of biopolymers, continuous microfluidics and heterogeneous living/controlled radical polymerizations [116]. This unique construction of NG enables them as a promising candidate in the area of drug delivery, diagnostics and imaging.

**Figure 5:**
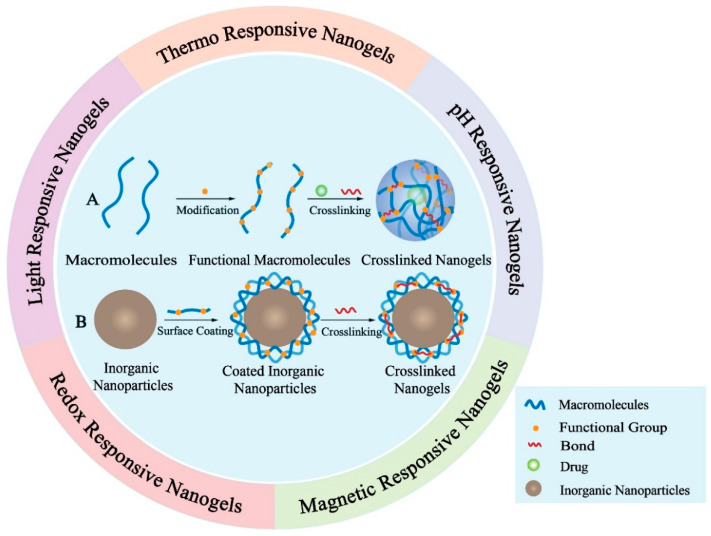
Schematic demonstration of nanogels. The inner circle illustrates the preparation of two representative crosslinked nanogels. (**A**) Preparation of macromolecules-based crosslinked nanogels; (**B**) Preparation of inorganic nanoparticles-based crosslinked nanogels. The outer circle demonstrates five typical stimuli-responsive nanogels. The drugs encapsulated will be released from nanogels after triggered by different stimulus like temperature, pH value, magnetic field, redox potential and light.

The NG-based drug delivery system possesses specific properties, such as large surface area and good structure stability, which endow NG with a high drug loading capacity and the ability to swell [117]. Notably, the higher surface area and the 3D network structure of NG enable the encapsulation of hydrophobic or hydrophilic drugs in their internal network, potentially protecting these drugs from degradation during storage or in circulation [118]. Unlike conventional NPs, NG possesses a tunable particle size and particle shape, sensitivity to pH, temperature, ionic strength, redox conditions and other external stimuli, providing them with effective controlled drug release capabilities. For example, some current studies of NG focus on the construction of stimuli-responsive systems based on the special conditions of the tumor microenvironment like low pH, high interstitial fluid pressure (IFP) and high GSH concentration to achieve targeted drug delivery and controlled release [119,120,121]. Moreover, NG can be designed as needed and their circulation time can be prolonged by surface modification. By the implementation of different kinds of polymers or inorganic NPs and various crosslinking chemical methods, it is possible to fabricate novel drug delivery systems based on NG with favorable characteristics [122] (Figure 5). Therefore, the NG-based drug delivery system has attracted more attention in recent years and will play an important role in current cancer therapy. Despite the merits mentioned above, several issues including uncontrollable size, variable morphology, and the lack of relevant clinical data relating to the safety and efficiency of nanogels in vivo still limit their clinical application. Hence, there is an urgent need for more relevant pre-clinical and clinical data of nanogels to speed up the clinical translation of them.

### 6.1. Encapsulation of Small Molecule Drugs with Nanogel

NG has made significant progress in the targeted delivery of small molecular drugs. Currently, small molecule drugs used in tumor therapy mainly contained chemotherapeutic drugs and photosensitizers, such as DOX, paclitaxel (PTX), curcumin and tetraphenylporphinesulfonate (TPPS), and so forth. Many researchers not only designed stimulus-responsive carriers, but also adopted multidrug combination therapy for synergistic effects. For example, DOX and cisplatin (CDDP) were the most widely clinically used chemotherapeutic drugs, which were confirmed synergistic effects for many malignancies [123]. A CDDP-crosslinked active targeting hyaluronic acid (HA) nanogel (CDDPHANG) was studied for effective DOX delivery to treat osteosarcoma. CDDP acted as a crosslinker to enable the drug to have a pH-responsive release capability as well as served as ancillary anticarcinogen to prevent premature release [28]. In vitro studies confirmed the optimized biodistribution and the targeted delivery of CDDPHANG/DOX. In addition, the in vivo results based on the xenografted osteosarcoma mouse model demonstrated the strong antitumor efficacy of CDDPHANG/DOX. Furthermore, the negligible organic injury also indicated the low toxicity of this nano delivery system, illustrating the great potential of CDDPHANG/DOX in clinical application.

When small molecule drugs enter cancer cells, they are usually trapped in endosomes or lysosomes, which significantly weakens the therapeutic efficacies of these drugs because the endo/lysosomal entrapment hinders their transportation to other target organelles. To address the above issues, Zhang et al. designed a novel delivery system based on pH-sensitive and size-changeable NG, which did not intend to avoid the endo/lysosomal entrapment problem but to utilize this entrapment phenomenon for retarding the efflux of nano drugs from drug-resistant cancer cells [29]. As ultrabright organosilica nanodots (OSiNDs) were reported to aggregate in the acidic lysosomes for long-term lysosome imaging, Zhang’s group prepared a pH-sensitive nanogel via the supramolecular self-assembly of OSiNDs, the biocompatible copolymer PEG-PLE, and the photosensitizer TPPS [124]. This TPPS-loaded NG could elude the drug efflux pumps and realize massive drug influx in multidrug-resistant A549/DDP cancer cells. Likewise, the small nanogel was found to enter the cells through endocytosis and then aggregated in the acidic endosomes/lysosomes to form larger particles, thus down-regulating their exocytosis from the cells. Furthermore, in vivo experiments based on A549/DDP tumor-bearing nude mice showed that the nanogels prolonged the retention of the photosensitizer, which was beneficial for the operation of repeated PDT with a single drug injection.

### 6.2. Encapsulation of Biological Macromolecules with Nanogel

Biomolecules, such as proteins and genes, are presently widely used as antitumor therapeutics. However, due to their large molecular weight, easy degradation property, and difficulty in passing through cell membranes, their efficient delivery is challenging [125]. Fortunately, NG is one of the potential carriers for these macromolecular biological drugs due to their excellent drug-loading capacity, targeting ability, stability, and hydrophilicity [114,125]. Wang et al. constructed an amphiphilic pH-sensitive galactosyl dextran-retinal (GDR) NG to effectively encapsulate OVA antigen to form GDR/OVA nanovaccine through self-assembly. In vivo results based on a xenografted melanoma mouse model showed that this GDR NG could not only promote DC maturation but also facilitate antigen uptake and cytosolic antigen release in DCs, indicating that DC-targeted pH-sensitive GDR NGs could be exploited to deliver macromolecular biological drugs [30]. siRNA exhibits great therapeutic potential in cancer diseases due to its ability to silence the genes [126]. Li et al. constructed a bioreduction-ruptured NG for switch on/off release of Bcl2 siRNA in breast cancer therapy. Thiolated PEI of 1.8 kDa was cross-linked with biodegradable dextrin to form bioreduction-ruptured dextrin NG (DSP) by disulfide bond for intracellular burst release of Bcl2 siRNA. When DSP/siRNA was uptaken into tumor cells, a high concentration of GSH in cytosol could degrade DSP NG into single PEI-1800 and dextrin with low toxicity followed by the controlled release of the packed siRNA [31]. A tumor suppression study of DSP based on 4T1-Luc tumor-bearing BALB/C mice also exhibited a superior antitumor activity, lower cytotoxicity, and almost no hemotoxicity in vivo.

## 7. Nanoemulsion

Nanoemulsions are colloidal dispersions consisting of oil, surfactant and an aqueous phase, where surfactants and co-surfactants form a stable coating over the dispersed droplets, mostly oil-in-water types [127]. Nanoemulsions are stabilized with emulsifiers which are amphiphilic so that they can decrease interfacial tension between two phases [128,129]. These pharmaceutical formulations are biocompatible and generally recognized as safe (GRAS) grade of excipients [130]. The structure and composition of nanoemulsions endow them with excellent properties of thermodynamic stability, small size distribution, high encapsulation capacity, as well as large surface area. In addition, nanoemulsions possess a high encapsulation efficiency for hydrophobic components, good physicochemical stability and enhanced bioavailability. They could also prolong drug circulation time and protect drugs against hydrolysis and oxidation [131]. Amazingly, nanoemulsions can be administered via a variety of routes, which broadens their therapeutic applications in cancer treatment. For instance, the oral administration of PTX using a nanoemulsion platform could protect drugs from degradation and avoid first-pass metabolism, which resulted in an increased transport of PTX, further enhancing the cellular uptake of PTX in breast cancer cells [132]. Localized injection of acai oil nanoemulsion exhibited superior photodynamic performance in melanoma treatment. High loading of acai oil in the nanoemulsion reduced tumor volume by 82% when irradiated with a light-emitting diode in tumor bearing C57BL/6 mice compared with the control group [133]. Hence, nanoemulsions have become a focus of research in targeted drug delivery because of their potential to achieve efficient therapeutic effects and minimize adverse effects. Various drugs encapsulated in nanoemulsions are designed and used to treat solid tumors including colon cancer, ovarian cancer, prostate cancer, breast cancer, lung cancer, melanoma as well as leukemia in pre-clinical studies [128]. Therefore, nanoemulsions are becoming an attractive dosage form and promising delivery platforms which can encapsulate both hydrophilic and hydrophobic molecules [134]. Despite the fact that nanoemulsions possess many advantages, no example of this kind of drug carrier has been approved by the FDA so far, which is a result of several obstacles that limit the successful clinical translation of this system. For example, the large-scale production of multifunctional nanoemulsions is an issue which needs to be solved at the current stage. In addition, the in vivo metabolism, as well as the long-term stability and safety of nanoemulsions, need to be carefully evaluated and studied.

### 7.1. Encapsulation of Small Molecule Drugs with Nanoemulsion

Ahmad and colleagues developed an oil-in-water nanoemulsion encapsulating the DHA-SBT-1214, a novel omega-3 fatty acid conjugated taxoid prodrug, against prostate cancer stem cells [32]. This nanoemulsion was composed of a chemotherapeutic drug SBT-1214 and polyunsaturated fatty acids (PUFAs) docosahexaenoic acid (DHA) to improve the cancer-specific toxicity. Among naturally occurring PUFAs, DHA exhibited the highest potency and has been studied extensively. For example, a DHA-paclitaxel conjugate Taxoprexin^®^ has demonstrated efficacy in Phase II clinical trials against prostate, breast, gastric and lung cancers, as well as metastatic melanoma [135]. Via the EPR effect, the DHA-SBT-1214 nanoemulsion was delivered to a PPT2 prostate tumor and could release SBT-1214 inside the cancer cells. Moreover, DHA has high affinity to bloodstream transporter human serum albumin, and thus could effectively reverse drug resistance and enhance the cytotoxic effect. In vitro studies based on PPT2 cells demonstrated the successful uptake and efficient delivery of DHA-SBT-1214 nanoemulsion to tumor cells. In vivo results obtained from PPT2 tumor xenografts mice model also showed that weekly intravenous administration of the DHA-SBT-1214 nanoemulsion led to a dramatic suppression of tumor growth compared to the control groups. Both in vitro and in vivo results indicated that this nanoemulsion possessed significant antitumor efficacy against prostate tumors.

Immunotherapy related to natural killer (NK) cells has garnered plenty of attention to overcome the clinical bottlenecks of the cancer treatment [136]. However, the immune response can be stunted by the tumor microenvironment such as secretion of transforming growth factor-beta (TGF-β). Herein Liu et al. established a nanoemulsion system (SSB NMs) to co-deliver the TGF-β inhibitor and selenocysteine (SeC) to enhance anticancer effects [33]. SeC was an essential amino acid that could enhance the immune activities of NK92 cells. In addition, previous studies have demonstrated TGF-β played a promoting role in tumor growth and malignant progression [137,138]. As a consequence, the SSB NMs could potentiate the immunity and cytolytic potential of NK92 cells. In vitro experiments towards NK92 cells suggested that SSB NMS could improve the cleavage ability and reduce the cytotoxicity. In vivo research using breast tumor-bearing nude mice demonstrated that SSB NMs significantly suppressed tumor growth by 47.02% compared to a control group, suggesting the high potency of NK cell-based cancer immunotherapy. Besides, the combined application of immune checkpoint (ICP) blockade and chemotherapeutic drugs have also been proposed as a promising strategy for tumor treatment [139]. For instance, Jia’s group constructed a pH-responsive pickering nanoemulsion (PNE) to deliver both ICP inhibitor HY19991 and DOX, which was termed D/HY@PNE [34]. HY19991 was a small molecule inhibitor, which could block the PD-1/PD-L1 interaction between T cells and tumor cells, resulting in the activation of T cells. PNE was a multifunctional system with pH-responsive, hydrophilicity–hydrophobicity switch, and redox-responding properties. With the hydrophilicity–hydrophobicity transformation in the acidic tumor microenvironment, D/HY@PNE released the ICP inhibitor HY and DOX. D/HY@PNE exhibited the internalization in vitro cellular uptake by murine breast 4T1 cancer cells. Other in vitro experiments also indicated that D/HY@PNE could enhance tumor penetration and induce immunogenic cell death. In addition, in vivo experiments based on 4T1 tumor-bearing mice showed the tumor damage and significant decrease in cell proliferation after treatment with D/HY@PNE, illustrating the promising potential of nanoemulsions to co-deliver ICP blockade and chemotherapeutic drugs to enhance antitumor efficacy. Therefore, the combination of immunotherapy and chemotherapy will become the research focus of cancer therapy in the future.

### 7.2. Encapsulation of Biological Macromolecules with Nanoemulsion

As mentioned above, immune checkpoint blockade (ICB) could block the PD-1 and PD-L1 axis to rejuvenate exhausted T cells, achieving remarkable tumor inhibitory effects [140]. However, its clinical translation is limited due to the inhibition of the tumor microenvironment and the side effect of antibody binding [141]. To address the issue, Zhang et al. developed a biomimetic nanoemulsion camouflaged with a PD-1-expressing cell membrane to co-deliver the photosensitizer perfluorocarbon and PD-1 protein (substituting for a PD-L1 antibody) against hypoxic breast tumors [35]. The perfluorocarbon of the nanoemulsion had a high affinity for oxygen, so it could transport a large amount of oxygen to hypoxic tumor regions and then generate singlet oxygen to kill tumor cells, serving as the source of PDT against hypoxic tumors. Via the EPR effect, the biomimetic nanoemulsion facilitated the accumulation of drugs at the tumor site to achieve the synergy effect of PDT and immunotherapy. In vitro studies confirmed the nanoemulsion could significantly enhance the cellular uptake and the oxygen concentration in tumor tissues. Moreover, cellular necrosis/apoptosis analysis further demonstrated the efficacy of inducing cell early apoptosis. In addition, in vivo experiments based on a unilateral subcutaneous 4T1 breast tumor-bearing mouse model showed that the biomimetic nanoemulsion could achieve a better tumor inhibition efficacy and significant PDT effect against hypoxic 4T1 cells. Overall, due to high biocompatibility, high stability and targeted tumor accumulation, the biomimetic nanoemulsions consisting of clinically used therapeutic agents represent a new and promising strategy in cancer therapy.

## 8. Hybrid Nanoparticle

**Figure 6:**
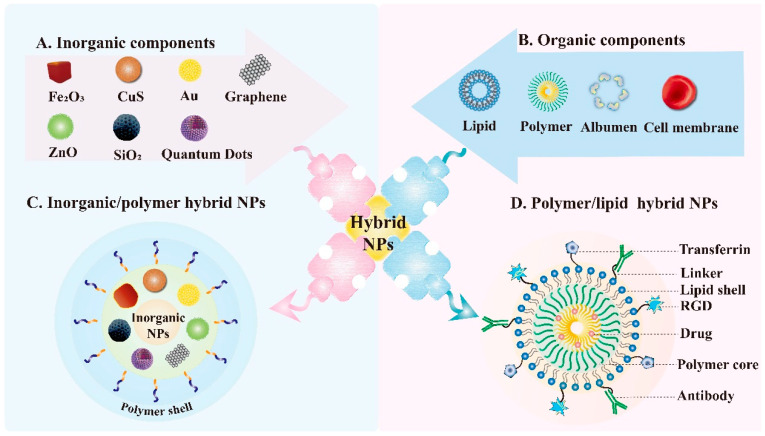
Schematic illustration of hybrid NPs. The representative inorganic nanomaterials (**A**) and organic nanomaterials (**B**) applied in hybrid NPs. (**C**) Composition of inorganic/polymer hybrid NPs. The combination of each type of inorganic core with the corresponding polymer results in hybrid NPs with different properties. (**D**) Structure of polymer/lipid hybrid NPs which consists of a polymer core encapsulating drugs and lipid shell. The lipid shell can also conjugate to a variety of targeting agents like antibodies, arginyl glycyl aspartic acid (RGD) and transferrin to ensure target delivery.

Generally speaking, hybrid materials refer to a mixture of two or more nanometer or micro-scale components. In most cases, one of the components of hybrid materials is inorganic, the other is organic matter in nature. Inorganic/polymer NPs and polymer/lipid NPs are two representative hybrid NPs that have been widely investigated [142,143] (Figure 6). The hybrid material is mixed at the micro-scale, and the interior is more uniform, so it does not show the characteristics between the two phases, but shows other novel characteristics [144]. The emergence of these novel materials provides a new idea for the construction of a nano-targeted drug delivery system. There are cavities in the hybrid NPs, which can contain small molecular chemotherapeutic drugs and biological macromolecular drugs, such as proteins and nucleic acids, according to the properties of different materials. Generally, drugs can not only be encapsulated by physical actions, such as intermolecular force, electrostatic adsorption and hydrogen bonding, but can also be coupled by chemical bonds [145]. In order to change the pharmacokinetic parameters, enhance the stability and solubility of the drug in vivo, as well as improve the adverse properties of the drug. Hybrid NPs are generally modified by materials with good biocompatibility or specific functions to achieve the target effect, which can also effectively prevent NPs from being swallowed by macrophages, prolong the circulation time of NPs in vivo, and improve the bioavailability of drugs. In addition, the tumor-targeting materials can be modified outside the NPs to enhance the aggregation of the drug in the tumor site and improve the efficacy [146]. In summary, owing to the common excellent properties of many materials, the hybrid NPs possess multifunctionality beyond a single material. In addition, hybrid NPs are easy to functionalize with other chemical groups, which broaden their modification possibilities. However, like other nano delivery systems, the preparation process of hybrid NPs is also complicated and the production cost is relatively high, which leads to the fact that the application of hybrid NPs still stays in the laboratory research stage.

### 8.1. Encapsulation of Small Molecule Drugs with Hybrid Nanoparticle

The modification of NPs with proteins is a powerful approach that can bring plenty of additional advantages to the NPs [147]. For example, protein coronae fabrication creates a type of biologically inert surface that endows the NPs with a stealth effect which prolongs the circulation time in the blood. Besides, the protein molecules on the NPs provide the biological identity and reduce the cytotoxicity of the nanocarriers and improve their biocompatibility. Hence, the synthesis of the polymer-protein core-shell NPs (CSNPs) is essential for drug delivery. Gold NPs (AuNPs) can be used for targeted cancer therapy due to their wonderful properties such as fascinating size-related electronic, optic, magnetic, and catalytic imaging ability [148,149]. Therefore, Hong et al. developed polymer-AuNP-protein CSCNPs with the poly(ε-caprolactone) (PεCL), which encapsulated curcumin (CUR) as hydrophobic cores, then adopted a Raman probe to decorate AuNP shells. The protein molecules in the coronae were functionalized with folic acid (FA) [36] and 4T1 cells were used to investigate the biological safety and antitumor effect of the hybrid NPs as drug carriers in vitro. The cellular uptake of the FA-containing CSCNPs by 4T1 tumor cells is much higher than that by the FA free CSCNPs while the survival rate of tumor cells was in reverse, which explained that the functionalization of CSCNPs with FA can significantly improve the internalization of NPs into 4T1 tumor cells due to the overexpressed folate receptors on the cells.

Ding et al. developed a novel hybrid nanomedicine (PEG-Au/FeMOF@CPT NPs), which was fabricated by the metal-organic framework (MOF) NPs and Au NPs as building blocks for cancer chemotherapy and chemodynamic therapy (CDT) [37]. Au/FeMOF NPs were used as vehicles to encapsulate camptothecin (CPT), of which the hybridization by Au NPs greatly improved the stability of the nanomedicine in a physiological environment. Besides, Au/FeMOF NPs could stimulate the production of ROS, which was beneficial to the synergistic antitumor effect of CDT. Triggered by the high concentration of phosphate inside the cancer cells, the new DDS could effectively collapse after internalization, resulting in the complete drug release and activation of the cascade catalytic reactions. HepG2 cells were used in vitro experiments to test the antitumor effect of the hybrid NPs, the results showed that the blood circulation time and tumor accumulation of the NPs were significantly increased due to the EPR effect and sophisticated fabrications. In vivo antitumor studies were performed using HepG2 tumor-bearing nude mice, demonstrating that the combination of chemotherapy and CDT effectively suppressed the tumor growth and avoided the systemic toxicity of this nanomedicine.

### 8.2. Encapsulation of Biological Macromolecules with Hybrid Nanoparticle

Prohibitin1 (PHB1) is a target protein that is associated with tumor suppression. High PHB1 tumor expression is connected with poorer overall survival in patients with non-small cell lung cancer (NSCLC), further indicating PHB1 as a therapeutic target [150]. siRNA plays an important role in current cancer therapy; however, effective systemic in vivo delivery of siRNA to tumors remains a formidable challenge. To solve this problem, Wang et al. brought out a robust self-assembly strategy by developing a novel NPs platform composed of a solid polymer/cationic lipid hybrid core and a lipid-PEG shell for systemic siRNA delivery [38]. The hybrid NPs had a small particle size and could efficiently encapsulate siRNA and control its sustained release. Moreover, the hybrid NPs exhibited long blood circulation, high tumor accumulation, effective gene silencing and negligible in vivo side effects. In vitro results based on NCI-H460 cells showed that the NPs could silence PHB1 and induce tumor apoptosis effectively. Meanwhile, in vivo PHB1-targeted experiments based on immunocompromised mice bearing NCI-H460 (human NSCLC) tumor xenograft demonstrated that the hybrids NPs treatment resulted in the significant suppression of tumor growth, and the average tumor weight in the NP (siPHB1) group was decreased by about 70% while other groups had no obvious decrease in tumor weight. H and E staining results further demonstrated that there was no noticeable histological change in the tissues from the heart, liver, spleen, lung and kidney between saline and NP (siPHB1) groups, indicating that the hybrid NPs had no organ toxicity.

## 9. Exosome

**Figure 7:**
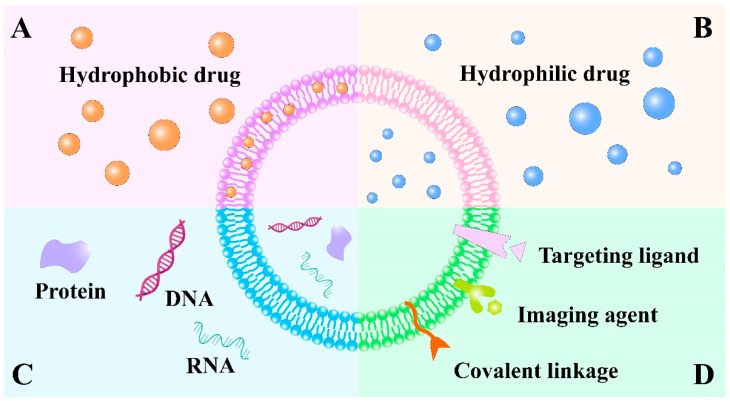
Schematic illustration of various types of exosome-based drug delivery systems. Exosomes are composed of a lipid bilayer that consists of lipids to enclose an aqueous core. Both the lipid bilayer and the aqueous core can incorporate (**A**) hydrophobic drugs and (**B**) hydrophilic drugs. (**C**) Exosomes can be used to deliver macromolecules such as DNA, RNA and protein. (**D**) Theranostic or imaging probes, specific targeting ligands and covalent linkage can also be attached to exosome surface for biomedical application.

Exosomes are naive extracellular vesicles, ranging in size from 40 to 100 nm, which are generated by all types of pro- and eukaryotic cells in the way of inward budding of the plasma membrane [151]. Structurally, exosomes are composed of a lipid bilayer to carry both hydrophobic and hydrophilic drugs [152] (Figure 7A,B). Considering the similarities between liposomes and exosomes, liposomes with selected components are commonly used to develop artificial exosome mimetics to overcome the limitation of low production yield of exosomes isolated from biological fluids or conditioned cell culture media [153]. Interestingly, exosomes contain a cargo of constituents of the parent cell, including DNA, RNA, lipids, metabolites and different receptors or surface adhesion proteins such as selectins, integrins, tetraspanins and CD18 receptors [154,155] (Figure 7C). Thus, the specific origin of exosomes possesses the unique property of homing selectivity to their donor cells. Importantly, exosomes are involved in inter- and intracellular communications and can transmit messenger molecules, such as messenger RNAs (mRNAs) and microRNAs (miRNAs), to target cells in the body. They transfer these substances to the recipient cells, which further affect the phenotype of the target cell and regulate its physiological functions [156,157]. In addition, theranostic or imaging probes, specific targeting ligands and covalent linkage can be attached to the exosome surface for biomedical applications [152] (Figure 7D). In general, exosomes released by tumor cells are involved in tumor progression and also prevent the accumulation of chemotherapeutic reagents, thus, tumor cells are commonly beyond the cell sources of exosomes. Exosomes derived from mesenchymal stem cells (MSCs) and DCs possess excellent intrinsic therapeutic activity and are used more widely [158]. Therefore, by virtue of their nanosize, low immunogenicity and specific surface proteins, exosomes and exosome-mimetics are considered a new generation of bioinspired-nanoscale DDS to carry various anticancer payloads such as siRNAs, proteins, chemotherapeutic agents and even imaging agents with a desired target [159].

### 9.1. Encapsulation of Small Molecule Drugs with Exosome

Exosomes or synthetic exosomes can transport classical chemotherapeutic agents to the tumor cells with reduced toxicity [160]. It was reported that DOX encapsulated in exosomes could increase its therapeutic index in breast and ovarian cancer mouse models [161]. To this aim, Wang et al. used the M1-exosomes (M1-Exos) derived from M1 macrophages as the carrier to deliver anticancer drug paclitaxel (PTX) to tumor tissues because M1 macrophages were reported to release pro-inflammatory factors and induce antitumor immune responses [39]. The macrophage cells were treated by the M1-Exos obtained from the IFN-γ-activated macrophages to detect associated signal pathways in vitro, the expression of NF-κB at mRNA level treated with higher concentration (40 μg/mL) increased compared with the control group (20 μg/mL), which indicated that M1-Exos can activate the NF-κB pathway in a dose-dependent manner. Moreover, M1-Exos can promote the production of cytokines to create a pro-inflammatory environment, and thus enhanced the antitumor effects of chemotherapy drugs to kill tumor cells. In the 4T1 tumor-bearing mice model, PTX-M1-Exos groups exhibited higher antitumor effect than the M1-Exos or PTX group alone, indicating that the enhanced therapeutic effect might be attributed to the combined function of M1-Exos by activating the NF-κB pathway and providing a local inflammatory environment.

Brain endothelial cell derived exosomes, which express more CD63 tetraspanins transmembrane proteins, are used as a targeted carrier by Yang et al. for anticancer drug delivery in the treatment of brain cancer [40]. With specific homing CD63 biomarkers, endothelial exosomes increased the uptake of fluorescent marker rhodamine 123 via receptor mediated endocytosis. The study proved that the endothelial exosomes tend to transport anticancer drugs across the blood brain barrier (BBB), leading to the significantly enhanced cytotoxic effect of DOX and PTX in U-87 MG brain cancer cells. Besides, an in vivo zebrafish study showed that exosomes delivering DOX were able to cross the BBB, which subsequently exerted cytotoxic efficacy against brain cancer.

### 9.2. Encapsulation of Biological Macromolecules with Exosome

Mesenchymal stem cells (MSCs) are commonly harnessed to produce exosomes, and MSCs-derived exosomes possess the therapeutic capacity by themselves through a paracrine mechanism [162,163]. The liver-specific microRNA-122 (miR-122) is a bona fide tumor suppressor, which has been found to play a critical role in liver biology and disease [164]. The downregulation of miR-122 is associated with hepatocellular carcinoma (HCC) development and progression in HCC cell models [165]. Furthermore, MiR-122 has been shown to be involved in sorafenib resistance in HCC [166]. A miR-122 expression plasmid was transfected into adipose tissue-derived MSCs (AMSCs) to obtain miRNA-modified AMSCs by Luo et al. [41]. AMSC-derived exosomes (122-Exo), secreted by miR-122-transfected AMSC, were capable of mediating miR-122 communication between AMSCs and HCC HepG2 cells through downregulating the expression of miR-122-target genes. Furthermore, the efficient inhibitory effect of sorafenib observed on 122-Exo-treated HCC cells was significantly increased, which indicated that 122-Exo could enhance the chemosensitivity of HCC cells. Moreover, in vivo results based on nude mice bearing HepG2 cells also demonstrated that 122-Exo can sensitize HCC cells to sorafenib and produce a significant antitumor effect.

As a potential tumor suppressor, miRNA-let-7a (let-7a) has been identified to be deregulated in various cancers [167,168]. Therefore, Ohno et al. used exosomes to deliver the let-7a to epidermal growth factor receptor (EGFR)-expressing breast cancer cells [42]. The GE11 peptide, which possesses a specific affinity to EGFR, was modified to exosomes for achieving specific EGFR targeting. Thus, let-7a-containing GE11-positive exosome can specifically bind to HCC70 breast cancer cells in RAG2^–/–^ mice after intravenous injection, and the tumor growth was remarkably suppressed in vivo, which also implied that efficient tumor therapy can be achieved by GE11-positive exosomes through delivering miRNA to EGFR-expressing cancerous tissues.

## 10. Conclusions and Outlook

To summarize, the tremendous potential of nanomedicine in cancer treatment is non-negligible. There is no doubt that nano-based DDS with increasing multifunctionality and versatility will exist in the future to provide more customized therapy according to the personalized request of patients. Encouragingly, various types of nanocarriers including a wide range of lipids, polymers of natural and synthetic origin, inorganic nanoparticles and cellular vesicles are currently being investigated by many researchers for targeted delivery of biomolecules and therapeutic agents with promising in vitro and in vivo efficacy. The encapsulation of cancer therapeutic agents, such as chemotherapy drugs, nucleic acid therapeutics, small molecule inhibitors, phototherapy agents and therapeutic antibodies through nano-formulations, possess plenty of advantages including increased bioavailability, controlled release capability, the ability to prevent degradation, and preferential delivery to the tumor site. Besides, more advanced and innovative nano-based delivery systems have also been developed to deliver drugs specifically to the tumor site based on their unique properties, such as stimuli-responsive ability and active targeting ability, thereby largely enhancing their therapeutic efficiency.

Although there are numerous attractive features of nanocarriers, several challenges still hamper the translation of these delivery systems from bench to bedside. For instance, the complex structural design of nano-based DDS restricts their clinical translation. The complicated system construction, tedious material preparation, manufacturing process, reproducibility and quality control make the potential pharmaceutical development of academic DDS more difficult towards clinical transformation. In addition, alternations in the physicochemical properties of the nanocarriers in systemic circulation, such as change in particle size, aggregation behavior and premature drug release, also limit their successful clinical application.

Despite the challenges mentioned above, with the deeper understanding of tumor biology and progress in the development of nanomaterials, obstacles, such as maintaining the stability of therapeutic agents, controlling their pharmacokinetics, reducing nanotoxicity and achieving targeted delivery, can be overcome to some extent. Furthermore, through the efforts of many scientists, it is convinced that nano-encapsulated delivery systems involve novel scientific routes with adequate advanced technologies will pave the way for their clinical evaluation. The advancement of DDS and their progressive applications will open a new era for the diagnostic methodologies of several cancer types as well as their targeted therapy.

## Figures and Tables

**Table 1:** Summary of various nanostructures to encapsulate therapeutic agents in anticancer targeted drug delivery.

Type of Nanostructures	Encapsulated Anticancer Agents	Targeting Type	Tumor Model	In Vitro or In Vivo Study	Therapeutic Efficacy	Ref.
**Liposome**	5-carboxy-8-hydroxyquinoline (IOX1) and doxorubicin	EPR effect	Murine colon cancer	In vitro and in vivo	Promote T cell infiltration and activity, reduce tumor immunosuppressive factors, elicit long-term antitumor immunological memory, decrease the tumor growth of 4T1 orthotopic and lung metastatic dual tumors, prolong the survival for over 80 days	[9]
	Indocyanine green	Dendritic lipopeptide-mediated active targeting	Murine breast cancer	In vitro and in vivo	Mitochondrion-targeted delivery, disrupt the mitochondrial membrane, generate ROS and eradicate tumor completely	[10]
	Platinum and verteporfin	Macrophage membrane protein-mediated active targeting	Murine breast cancer	In vitro and in vivo	Decrease the hypoxic region by 78.4%, relieve tumor hypoxia, inhibit local tumor growth, suppress lung metastasis and prolong animal survival to 43 days	[11]
	Cytochrome C and p53 proteins	Folic acid-mediated active targeting	Human breast cancer	In vitro and in vivo	Specific delivery of bio-macromolecule drugs and inhibit tumor growth	[12]
	Catalase	EPR effect	Murine breast cancer and prostatic patient-derived xenograft (PDX) tumor	In vitro and in vivo	Improve tumor oxygenation, promote infiltration of CTLs and inhibit tumor growth	[13]
**Reconstituted high-density lipoprotein (rHDL)**	Gambogic acid	Apo A-I-mediated active targeting and pH-sensitive targeting	Human liver cancer	In vitro and in vivo	Display approximately 5-fold increase in cytotoxicity compared to free GA, attain superior tumor accumulation and significant inhibition of tumor growth in vivo	[14]
	Valrubicin	Apo A-I-mediated active targeting and magnetic targeting	Human prostate cancer	In vitro and in vivo	The cytotoxicity toward PC-3 cells is 4.6 and 31 times more effective at the respective valrubicin concentrations of 42.4 µg/mL and 85 µg/mL than valrubicin alone, induce tumor cell apoptosis, effectively enhance the therapeutic efficacy	[15]
	Cholesterol-conjugated siRNA	Apo A-I-mediated active targeting	Human breast cancer	In vitro and in vivo	Efficiently decrease VEGF expression level by about 54.4% and inhibit the formation of intratumoral microvessels at the tumor tissue	[16]
	miR-204-5p inhibitor	Apo A-I-mediated active targeting	Human ovarian cancer	In vitro and in vivo	Silence the expression of the oncogene miR-204-5p at tumor sites, inhibit tumor growth, induce 50% reduction in tumor weight	[17]
**Micelle**	Artesunate	pH-sensitive targeting	Murine colon cancer	In vitro and in vivo	Inhibit original tumor growth, the tumor volumes in micelle group are 1.34-fold smaller than ART group in the 21st day post-treatment	[18]
	S-nitrosoglutathione and doxorubicin	ROS and GSH-sensitive targeting	Human liver cancer	In vitro	Reverse chemo-resistance of hepatocellular carcinoma and selectively kill cancer cells, show a 14-fold increase in the uptake of DOX, enhance the tumor cells internalization of NO and DOX	[19]
	Melphalan	Reduction-responsive targeting	Human retinoblastoma	In vitro	Enhance the cytotoxicity against Rb tumor cells	[20]
	Gemcitabine and MiR-519c	Redox-responsive targeting	Murine pancreatic ductal adenocarcinoma	In vitro and in vivo	Decrease HIF-1α expression, reverse the GEM resistance and inhibit the tumor growth	[21]
	siRNA-PD-L1	pH-sensitive targeting and antibody-mediated active targeting	Murine melanoma	In vitro and in vivo	Silence the expression of PD-L1 protein, induce tumor cell apoptosis, prolong the survival time of mice to at least 9 days	[22]
**Dendrimer**	Doxorubicin	pH-sensitive targeting	Human ovarian and breast cancer	In vitro and in vivo	Penetrate deeper in tumor tissues, inhibit the tumor growth	[23]
	Pyropheophorbide-a	Light-sensitive targeting	Murine breast cancer	In vitro and in vivo	Induce efficient induction of ROS production and significant inhibition of tumor growth	[24]
	Camptothecin	ROS-responsive targeting	Murine pancreatic ductal adenocarcinoma	In vitro and in vivo	Possess a high efficiency of active tumor penetrating capability and antitumor effect, the dendrimer group exert an average tumor inhibition rate of 90.2%	[25]
	Immunostimulants	Antigen-mediated active targeting	Human breast cancer	In vitro and in vivo	Promote the immune reaction, produce related antibody, inhibit the tumor growth	[26]
	Toxin protein saporin	GSH-response targeting	Murine breast cancer	In vitro and in vivo	Efficiently inhibit the tumor growth	[27]
**Nanogel**	Doxorubicin	Hyaluronic acid-mediated active targeting	Human osteosarcoma	In vitro and in vivo	Prolong circulation time to about 60 h, reduce side effects, and enhance 1.4 times antitumor efficacy than that of free drugs	[28]
	TPPS	pH-sensitive targeting	Human lung cancer	In vitro and in vivo	Reduce drug efflux, increase drug uptake, inhibit autophagy and enhance antitumor effect	[29]
	OVA antigen	pH-sensitive targeting	Human melanoma	In vivo	Promote DC maturation, enhance antigen uptake, significantly enhance CD4^+^ T cell proliferation by 2 folds, increase tumor-specific IFN-γ production over 5 folds compared with soluble OVA	[30]
	Bcl2 siRNA	GSH-sensitive targeting	Murine breast cancer	In vitro and in vivo	Exhibit a superior antitumor activity, lower cytotoxicity, and almost no hemotoxicity, downregulate Bcl2 protein by about 70%	[31]
**Nanoemulsion**	DHA-SBT-1214	EPR effect	Human prostate cancer	In vitro and in vivo	Induce superior regression and tumor growth inhibition	[32]
	TGF-β inhibitor and selenocysteine	EPR effect	Murine breast cancer	In vitro and in vivo	Potentiate the immunity and cytolytic potential of NK92 cells, and increase tumor inhibition ratio up to 78.15%	[33]
	ICP inhibitor HY19991 and doxorubicin	pH-sensitive targeting	Murine breast cancer	In vitro and in vivo	Enhance tumor penetration, induce immunogenic cell death, and enhance antitumor efficacy of about 71% tumor growth inhibition rate	[34]
	Perfluorocarbon and PD-1 protein	PD-1-expressing cell membrane-mediated active targeting	Murine breast cancer	In vitro and in vivo	Enhance the oxygen concentration, induce cell early apoptosis, and achieve significant PDT effects	[35]
**Hybrid NPs**	Curcumin	FA-mediated active targeting	Murine breast cancer	In vitro	Decrease the survival rate of tumor cells	[36]
	Camptothecin	EPR effect	Human liver cancer	In vitro and in vivo	Effectively suppress the tumor growth and avoid systemic toxicity, the inhibition ratio is 85.6%	[37]
	Prohibitin1 siRNA	EPR effect	Murine lung cancer	In vitro and in vivo	Silence PHB1 and induce tumor apoptosis effectively	[38]
**Exosome**	Paclitaxel	M1-macrophages-mediated active targeting	Murine breast cancer	In vitro and in vivo	produce pro-inflammatory cytokines and potentiate the anti-tumor effects of paclitaxel	[39]
	Doxorubicin and paclitaxel	Exosome-mediated-active targeting	Human glioma	In vitro and in vivo	Deliver drugs across the BBB and exert cytotoxic efficacy against brain cancer	[40]
	Sorafenib	Exosome-mediated-active targeting	Human liver cancer	In vitro and in vivo	Increase the sensitivity of HCC cells and enhance the antitumor efficacy of sorafenib	[41]
	miRNA-let-7a	GE11 peptide-/epidermal growth factor-mediated active targeting	Human breast cancer	In vitro and in vivo	Bind to tumors specifically and suppress tumor growth	[42]

## Data Availability

Not applicable.
